# Synthesis and Characterization of a Semiconductor Diodic Bilayer PbS/CdS Made by the Chemical Bath Deposition Technique

**DOI:** 10.1021/acsomega.3c10051

**Published:** 2024-05-28

**Authors:** Abraham Encinas-Terán, Horacio A. Pineda-León, María R. Gómez-Colín, Laura R. Márquez-Alvarez, Ramón Ochoa-Landín, Alejandro Apolinar-Iribe, Sandra L. Gastélum-Acuña, Temístocles Mendívil-Reynoso, Santos J. Castillo

**Affiliations:** † Departamento de Ingeniería Química y Metalurgia, Universidad de Sonora, Blvd. Luis Encinas y Blvd. Rosales S/N Apartado Postal 626, Hermosillo, Sonora C.P. 83000, Mexico; ‡ Departamento de Matemáticas, Universidad de Sonora, Hermosillo, Sonora C.P. 83000, Mexico; § Departamento de Física, Universidad de Sonora, Hermosillo, Sonora C.P. 83000, Mexico; ∥ Departamento de Ingeniería Ambiental, Universidad Estatal de Sonora, Hermosillo, Sonora C.P. 83100, Mexico; ⊥ CONAHCYT-Departamento de Investigación en Física, Universidad de Sonora, Hermosillo, Sonora C.P. 83000, Mexico; # Departamento de Investigación en Física, Universidad de Sonora, Hermosillo, Sonora C.P. 83000, Mexico

## Abstract

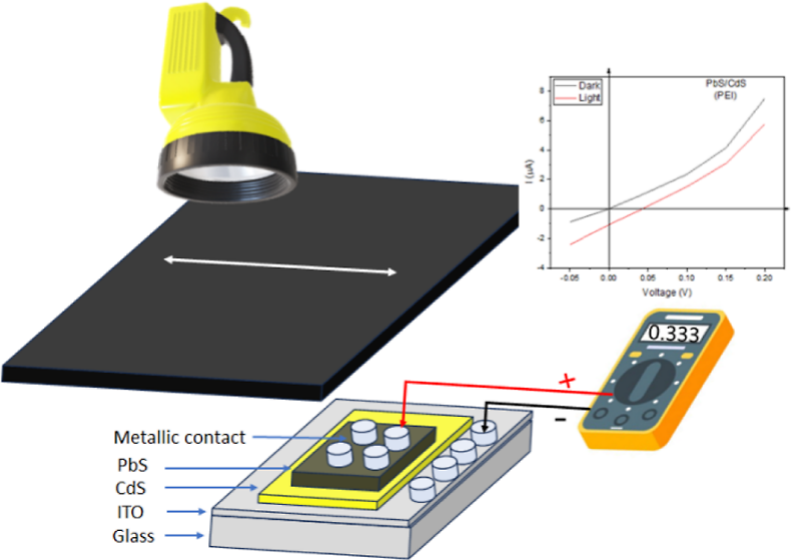

In this work, we report a heterojunction formed by a PbS/CdS bilayer using the chemical bath deposition (CBD) technique because it is a relatively simple, fast, and low-cost technique; is permitted to obtain high-quality thin films (TFs); and also covers large areas. Some characterizations have been carried out to confirm the identity of the involved bilayer. For the cadmium sulfide (CdS) film, optical properties such as absorption, transmission, reflection, extinction coefficient, and refractive index were measured. Moreover, the bandgap was calculated, and morphology was obtained by scanning electron microscopy (SEM). Also, X-ray diffraction (XRD) and high-resolution transmission electron microscopy (TEM) were performed for the synthesis of CdS films. On the other hand, for the synthesis of lead sulfide (PbS) films, we performed TEM, energy-dispersive spectroscopy, and XRD. A surface morphological SEM image of the PbS film synthesized was also taken. The multiheterojunction PbS/CdS bilayer was characterized by the current–voltage (*I*–*V*) curve, and the behavior of the bilayer was evaluated under the conditions of darkness and controlled fixed lighting, detecting a very slight photosensitivity of the complete diodic device through those measurements. The calculated bandgap for the CdS TF was *E*_g_ = 2.55 eV, while after a chosen thermal annealing, the bandgap decreased to 2.38 eV. On the other hand, the PbS film presented a cubic structure.

## 1 Introduction

Semiconductors are materials with electrical conductivity σ in the range of 10^–10^ < σ < 10^4^ (Ω cm)^−1^. However, the electrical conductivity of a conductor material is σ > 10^5^ (Ω cm)^−1^ and that of an insulator material is σ < 10^–10^ (Ω cm)^−1^. Typical values of electrical conductivity, σ, are determined primarily by the concentration of free electrons, n, in each type of material. Semiconductors have a bandgap between 0 and 4 eV for electrons. Metals or semimetals have a bandgap of 0 eV, whereas insulators have a bandgap larger than 4 eV. Semiconductor materials can also be classified based on different criteria. According to the number of different atoms present in semiconductor materials, they are classified as elementary (Si, Ge, Se, etc.), binary (GaAs, CdTe, ZnS, ZnO, CdS, etc.), ternary (CdZnTe, CuInSe, CuInS, CdHgTe, etc.), quaternary, etc., semiconductors. Depending on the location in the periodic table of the atoms that make up a semiconductor, they are classified as Group IV semiconductors, Si, Ge, etc.; Group III–V semiconductors, GaAs, InSb, InP, etc.; Group II–VI semiconductors, CdS, CdTe, ZnO, etc.; Group IV–VI semiconductors, PbS, PbTe, SnTe, etc.; and Group II–V semiconductors, CdAs, etc.; among others. Semiconductors are materials that exhibit the properties of both an insulator (like glass) and a conductor (like metals) under certain conditions in their operating environment. They have a crystalline structure due to the bond, just as they contain a few free electrons at room temperature. The electrical conductivity, σ, of semiconductors increases when the temperature raises, which is contrary to the behavior of metals. Semiconductors that are undoped or contain pure elements are called intrinsic semiconductors. Semiconductors that may be doped or contain impurities in excess are called extrinsic semiconductors. When the impurities are mostly free holes, the semiconductors are called p-type extrinsic semiconductors, and when the impurities are mostly free electrons, the semiconductors are called n-type extrinsic semiconductors. Semiconductor materials are very versatile since their electrical properties can be varied by orders of magnitude relatively easily. They are a basic element in the field of science and technology. A good number of devices base their operation depending on the properties of semiconductor materials. Some of the devices are diodes, solar cells (SCs), photodetectors, transistors, thermistors, light-emitting diodes (LEDs), and so on. These properties enable them to have a wide range of applications in electronic and optoelectronic devices, such as diodes. For instance, zinc oxide (ZnO)-based LEDs have been developed through research,^1^ rare-earth free tunable LEDs,^2^ organic light-emitting diodes (OLEDs) like gallium-doped ZnO thin films (TFs),^3^ ethoxylated polyethylenimine (PEIE) and ZnO at PEIE-ZnO as functional layers for OLEDs,^4^ ZnO:Gd^3+^/Yb^3+^ as an emissive layer in LEDs,^5^ perovskite LEDs (PeLEDs),^6^ PeLEDs utilizing ZnO as an electron-transporting layer (ETLs)m,^7^ and AlGaN like deep ultraviolet LEDs,^8^ among others. Semiconductors are also used as potentiometers in the automotive sector,^9^ as a portable semiconductor refrigeration device,^10^ as well as in thin-film transistors (TFTs), which are of high interest for further investigation and application due to their ease of fabrication.^11^ Flexible oxide TFTs are used as memristive devices,^12^ p-type tellurium (Te) TFTs,^13^ indium oxide (In_2_O_3_)-based TFTs,^14^ and TFTs based in metal oxide semiconductors in emerging applications such as flexible–stretchable devices; biosensors; zinc tin oxide, indium gallium zinc oxide, indium tin zin oxide, nitrogen-doped indium tin oxide, and indium tin oxide (ITO) TFTs as promising biosensing devices; and so on as next-generation TFTs, among others.^15−22^ Solar energy is a freely available renewable energy source. Moreover, it is eco-friendly and can provide energy at a low cost. Crystalline and polycrystalline silicon TFs are very expensive for modular SCs, an alternative to low-cost TF semiconductors and to have the ability to perform better. For example, copper indium gallium selenide (CIGS) and cadmium telluride (CdTe) TF SCs are of low cost and have better efficiency than the amorphous silicon (α-Si) TF SCs.^23^ Metal chalcogenide TFs are grown using a different technique; some types of films are metal sulfide, metal selenide, and metal telluride, which show good quality and flexible substrates, among others, and have potential for photovoltaic cells.^24−27^

These devices have given rise to important technological advances that have had numerous applications in various fields of science such as engineering and medicine, as well as in everyday life, making semiconductors highly sought-after materials today and are also under constant development. They are extensively researched for expanding their potential applications, enhancing their performance, and innovating new technologies.

Semiconductor TFs have an extraordinary impact due to possibilities made with the different methods and synthesis techniques; moreover, with control, changes, or slight variation in their synthesis or growth processes, it is possible to obtain semiconductor TFs with significant improvements according to their potential applications. By knowing their manufacturing methods, as well as the physics of semiconductors, it will be possible to carry out far-reaching scientific and technological applications. TF deposition techniques are mainly physical and chemical methods as well as the physico-chemical method. Physical or chemical methods are based on the principle that causes film deposition.

The TF deposition technique by chemical methods includes electrodeposition, chemical spray pyrolysis (CSP), chemical vapor deposition (CVD), photo CVD, plasma-enhanced CVD, successive ion layer adsorption and reaction (SILAR), low-pressure chemical vapor deposition (LP-CVD), metal–organic vapor deposition (MOCVD), anodization, dip coating, sol–gel, and chemical bath deposition (CBD), among others.^28−37^

CBD also includes as chemical solution deposition (CSD), solution growth technique, controlled precipitation, or chemical deposition (CD). In 1835, J. Liebig first reported about the deposition of silver (Ag) films using CD (). CBD is one technique for producing films of solid inorganic or nonmetallic compounds on substrates by immersing the substrate in a precursor solution (an aqueous liquid). The CBD technique requires a vessel to contain the solution (aqueous solution with a few chemicals), a substrate on which deposition takes place (for example, glass, plastic, alumina, silicon, n-Si, sapphire, and so on), a thermostated water bath at a constant temperature, a mechanism to stirring (a magnetic stirrer or an ultrasonic bath), a thermometer, and an extraction hood. Properties of films are controlled with the concentration of chemical precursors, pH reaction solution, temperature (room temperature to usually <100 °C), and deposition time. The advantages of the CBD technique are as follows: it includes multiple substrate coating, covers a large area, has a wide range of substrates, shows fast performance, works at a relatively low temperature, does not include a vacuum system, does not have special equipment, is expensive, is simple to use, and requires reproducible samples and high-quality TFs. CBD is used to deposit films of different semiconductors. In 1869, it was reported that the first binary compounds of semiconductor films resulted in the formation of “lüsterfarben” on different metals from thiosulfate solutions of lead acetate, copper sulfate, and antimony tartrate, forming films of PbS, Cu–S, or Sb–S. A few years later, in 1884, it was reported that PbS films were prepared by reaction between thiourea (thiocarbamide) and alkaline lead tartrate. After that, in 1906, it was reported that infrared (IR) photoconductivity was observed in PbS. Years later, PbS and PbSe films were studied for IR detectors. In 1961, it was reported for the first time that the synthesis of CdS films was via CBD (in 1912, CdS was noted in the thiosulfate solution). In 1978, it was reported that CdS was used in CBD for photovoltaic cells.^38−41^ In the beginning of 1980s, different materials began to be deposited using the CBD technique, e.g., sulfides, selenides of various metals, some oxides, and some ternary compounds, among others, and today, semiconductor films have been increased by the CBD technique.

There exist many research works related to semiconductors made by CBD and its applications.^42−46^

There is also a growing interest in developing more efficient and cost-effective SCs to take advantage of areas with high solar radiation and to expand the market for better utilization of this natural resource in lieu of fossil fuels that generate pollution and are becoming increasingly scarce, as petroleum is a primary raw material for many essential materials. Although silicon SCs are highly efficient, they require a substantial infrastructure to manufacture and must be produced in a highly hygienic environment due to the reaction involved, as well as significant energy cost. Thus, many researchers have been investigating various other semiconductors for potential use in SCs, which offer a promising avenue for sustainable energy production.^47−52^

### 1.1 PbS and CdS in TFs

Lead sulfide (PbS) is a semiconductor IV–VI chalcogenide. PbS is an inorganic compound formed by lead and sulfur, which is abundantly found in nature, and its mineral form is known as galena; it is grayish black in color. PbS has a narrow direct bandgap (approximately 0.41 eV, for bulk) at room temperature, 300 K, and it has a large exciton Bohr radius of about 18 nm (exciton Bohr radius, for bulk). Bulk PbS has a cubic crystal structure, high dielectric constant, and high carrier mobility.^53−55^ A Bohr radius of 18 nm provides a high quantum confinement for electrons and holes, which allows increasing the absorption for solar radiation in the near-IR electromagnetic spectrum. PbS has high absorption within the ultraviolet visible (UV–vis) spectral region and has high transmittance within the IR spectral region, which allow its use in IR detectors.^56−61^ PbS has extraordinary optical and optoelectronic properties that are principally useful to mention, and some are for IR detection, SCs, photoresistance, diode laser, solar absorbers, quantum dots, transistors, and so on. Its use in the industry has been extensive, and primarily, it was used as a semiconductor in IR detectors due to its ability to absorb the IR spectrum. PbS TFs, although they block sunlight by absorbing a significant portion of it, allow the passage of the IR spectrum and are widely used in detectors. Some investigations have found that the TFs of PbS produced by CBD can be applied to SCs due to their photosensitive and photoelectric properties.^62^ Besides, some researchers achieved uniform TF coatings over a large area of PbS on fluorine-doped tin oxide (FTO) conductive substrates at various temperatures. The solar control properties of pyrolytically deposited PbS TFs have been investigated.^63^ PbS has also been used in nanoparticle form to achieve higher efficiencies, which is utilized in quantum dots within an inverted structure.^64^

Cadmium sulfide (CdS) is a metallic sulfide II–VI semiconductor chalcogenide. CdS is composed of cadmium and sulfur. Despite its primary usage as a yellow pigment, it is the most studied group II–VI metal chalcogenide for photovoltaic applications. CdS has a direct wide bandgap (approximately 2.4 eV) at room temperature, 300 K. It has mainly two crystalline phases, hexagonal wurtzite and cubic zinc blende structure. Its high transmittance and bandgap (2.4 eV) make it suitable for the fabrication of SCs, especially as an efficient window layer. Additionally, it has low resistivity, n-type conductivity, easy ohmic contact, high electrical conductivity, high electron affinity, and high gain photoconductivity. It is useful as an n-type transparent window material; it has high efficiency in TF SCs on cadmium telluride (CdTe), copper indium diselenide (CuInSe2), and so on. Ongoing research focuses on exploring additional properties and characteristics, especially in transparent TFs. CdS is an n-type semiconductor, and when alloyed with other metallic compounds, it is used in the production of fuses. CdS has excellent structural, physical, optical, and optoelectronic properties and can be used as a TF in photoconductive devices, photodetectors, photoresistors, radiation detectors, sensors, LEDs, active layers in TFTs, SCs, and so on. One of the main applications of CdS is in SCs.^65−74^

The CdS/PbS-quantum dot (QD) heterojunction is developed for SCs, where CdS acts as the n-type window layer. These SCs exhibited a high open-circuit voltage of approximately 638 mV and a short-circuit current density of 12 mA/cm^2^ 2, resulting in an efficiency of 3.3%.^75^ CdS serves as the window layer and PbS serves as the absorber layer in the CBD technique as CdS/PbS possesses high useful properties in SC manufacturing.^76^

CdS/PbS-type structures have been studied extensively by other authors. The theoretical maximum efficiency for photovoltaic conversion to generate energy from sunlight by a TF solar panel has not been reached. The CdS/PbS heterostructure can reach a maximum theoretical efficiency of 4.13%.^77^ If the grain size is reduced until it can be treated as a quantum dot, it is possible that the bandgap will increase, as well as the efficiency.^78^ Furthermore, the p–n heterojunction for light detection through photodiode devices is an important understudied application in semiconductor TFs.^79^ On the other hand, the interest still to fabricate CdS/PbS devices via the CBD method is due to low cost, versatility in the substrates, and scalability to large areas, and the obtained TF is of high quality. In this sense, it has been studied to vary the concentration of Cd and Pb in the CdS/PbS multilayers, heating the substrates, adding impurity concentration in CdS, varying the thickness in PbS (due to a Bohr radius of 18 nm), and so on.

CdS/PbS multilayers obtained by spray pyrolysis were studied by varying the concentration and molar ratio of the Cd and Pb salts and the number of layers; CdS/PbS multilayers were suitable for thermo-reflective films for solar control coating.^80^ On the other hand, n-CdS obtained by magnetron sputtering and PbS prepared via CD based on the p–n junction were designed to obtain the Al/PbS/CdS/ITO/glass heterojunction for photovoltaic cells.^81^

Using the CBD technique, the stack TFs of PbS/CdS and CdS/PbS were studied; the results exhibited bandgap tuning from their binary compounds (CdS and PbS) which are useful in solar technology, selective surface, and optoelectronic devices.^82^

The effect of the thickness in the CdS/PbS heterojunction obtained by the CBD method was studied; from the result of the photoresponse, it was deduced that the film thickness influences the efficiency of the SC.^83^ The effect of thickness of the PbS bilayer as an absorber layer was evaluated in the ITO/CdS/PbS/Au heterojunction via spray pyrolysis; the result showed that the efficiency of SCs depends on the thickness of PbS (absorber layer).^84^

Glass/ITO/CdS/PbS/C device layers were studied for photosensor application at low voltage.^79^

The CdS/PbS heterostructure obtained by the CBD technique was studied with different heat treatments; heat treatment has an impact on the morphology and electrical properties of cadmium lead sulfide TFs because annealing is important in developing ternary and quaternary semiconductors.^85^

In this work, a device that behaves like a diode operating under dark conditions and shows a photosensitive behavior when operating under controlled lighting conditions is elaborated. This device consists of an ITO-coated glass substrate, on which a cadmium sulfide semiconductor film is deposited, followed by a second lead sulfide semiconductor film, back contact/PbS/CdS/ITO/glass. The semiconductor films involved were synthesized by CBD, with formulations previously reported in the scientific literature.^86,87,89−91^

## 2 Experimental Section

To elaborate the multiheterojunction (MH), Ag/PbS/CdS/ITO/glass, chemical formulations (recipes) were used as previously reported.

The experimental formulation to synthesize the cadmium sulfide film was carried out at a temperature of 40 °C. A mix of 10 mL of cadmium dichloride at 0.05 M was used as a source of cadmium, followed by 20 mL of sodium citrate at 0.5 M, 5 mL of potassium hydroxide at 0.3 M, 5 mL of borate buffer at pH 10, and 10 mL of thiourea at 0.5 M as a source of sulfur and finally 40 mL of triple-distilled water. The reactions took place in precipitation flasks; three substrates were added to each flask so that the reaction was not too saturated, and the films were uniform. The reaction time was 3 h.

The experimental formulation to synthesize the lead sulfide film was carried out at a temperature of 70 °C. The following formulation is used: 5 mL of 0.5 M lead acetate (as a lead source), 4 mL of 2 M sodium hydroxide, 4 mL of polyethylenimine (PEI), and 4 mL of 1 M thiourea (as a sulfur source). The PEI concentration is achieved in such a way that each 50 mL of water had 3.5 mL of PEI. The reaction time was 20 min.

For characterization, an UV–vis Ocean Optics 4000 spectrophotometer, an X-ray diffractometer (D2 PHASER BRUKER), and a Phenom ProX Desktop SEM were used; on the other hand, the electrical measurements were carried out with an array developed at our laboratories. Additionally, transmission electron microscopy (TEM) studies of the samples were realized on a JEOL JEM-2010F instrument.

## 3 Results

**Figure 1:**
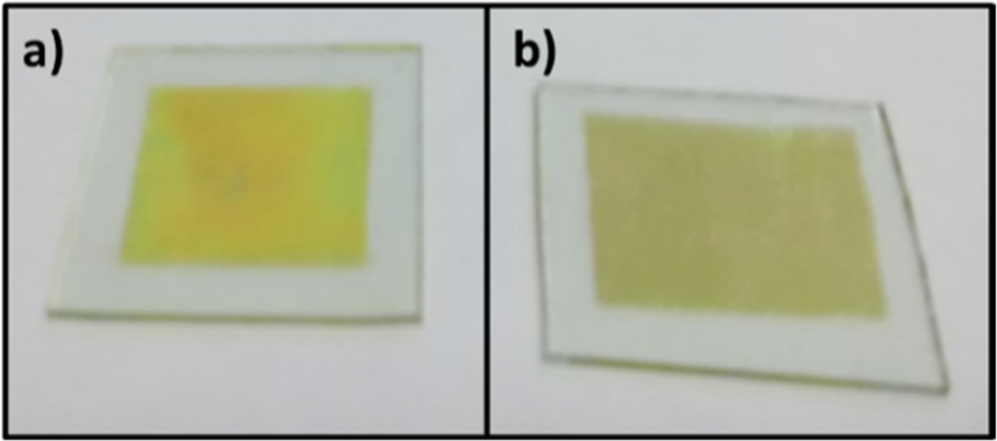
Photographs of CdS TFs on coated ITO glass substrates: (a) as ground and (b) with thermal annealing.

This section corresponds to the characterization results obtained from the semiconductor layers comprising the diodes fabricated in this research. Figure 1 shows the photographs of CdS TFs deposited on a glass substrate coated with ITO, Figure 1a shows the CdS film without thermal annealing, while Figure 1b shows the CdS film with thermal annealing for 5 min at 250 °C.

The deposited film without thermal annealing presented a better transparency and a slightly yellow color, while the thermal annealing film changed to a slightly greenish color; this last film was used for the elaboration of the planar extended diode.

**Figure 2:**
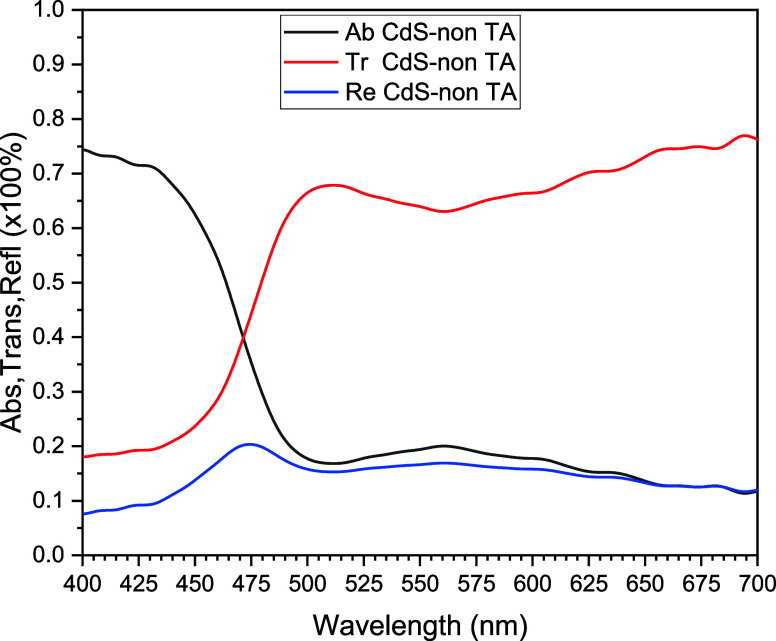
Three optical properties of the CdS TF, deposited on an ITO/glass substrate, without thermal annealing.

Following the plot, Figure 2 depicts three optical properties of the CdS TFs in the ground state, without thermal annealing used in this work; these properties are absorption, transmission, and reflection. These parameters are related through the following expressions
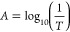
1
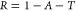
2

As can be seen, the behavior of the optical transmission is complementary to the behavior of optical absorption; moreover, the reflection or reflectivity property completes the conservation of electromagnetic energy that is incised on the material, CdS, in consideration. The optical properties of the material, reflection, transmission, and absorption in CdS are observed in Figure 2. Electromagnetic waves strike the CdS, and part of their energy is reflected, transmitted, and absorbed by CdS.

**Figure 3:**
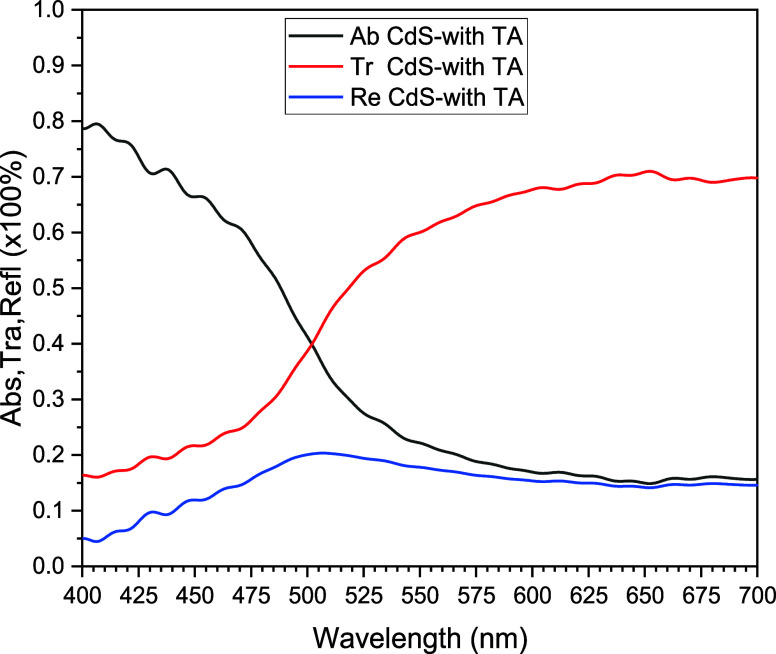
Three optical properties of the CdS TF, deposited on an ITO/glass substrate, with thermal annealing.

Figure 3 shows the optical properties of the CdS TFs, with thermal annealing at 250 °C for 5 min. The optical properties analyzed with thermal annealing are absorption, transmission, and reflection, which are related through eqs 1 and 2. For the CdS film deposited on ITO/glass, a similar behavior was obtained for its three fundamental optical properties; note that the behavior of the optical transmission and the behavior of the optical absorption are complementary, and the optical reflection completes electromagnetic conservation energy.

**Figure 4:**
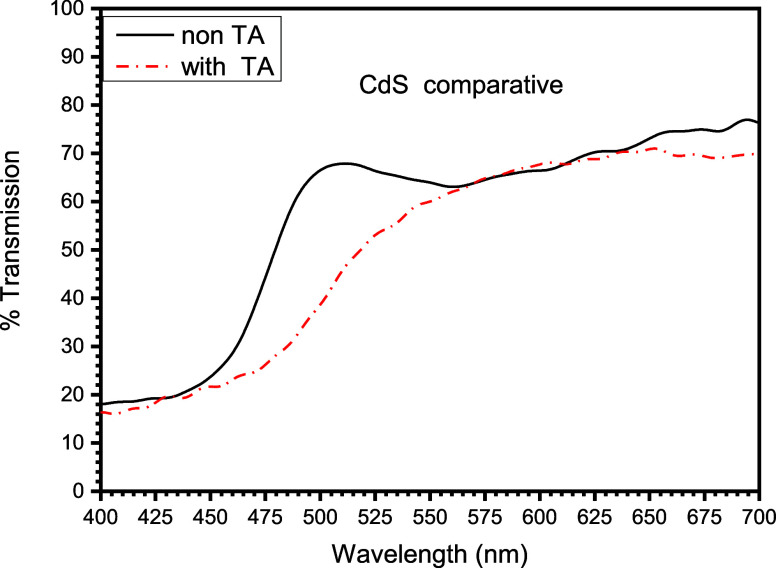
Comparative plots between transmissions of CdS TFs with and without thermal annealing.

In order to obtain a better appreciation of the comparison between the transmission behaviors of both with and without thermal annealing of the N-type semiconductor material, i.e., CdS, Figure 4 is constructed. Here, it shows a comparative graph between the transmissions of TFs of CdS, one of them without thermal annealing, while the other was subjected to a 250 °C thermal annealing for a time period of 5 min. As can be observed, the edge in the transmission is presented abruptly in the film without thermal annealing, reaching an approximate percentage of 70% for a wavelength of 445 nm, while the film with thermal annealing behaves with a soft transmission edge, displaced toward a higher wavelength of 480 nm.

Other characterizations made of CdS films were calculated from previous data, mainly from optical absorption, including extinction coefficients, light penetration depth, refractive index, and bandgap energies.

**Figure 5:**
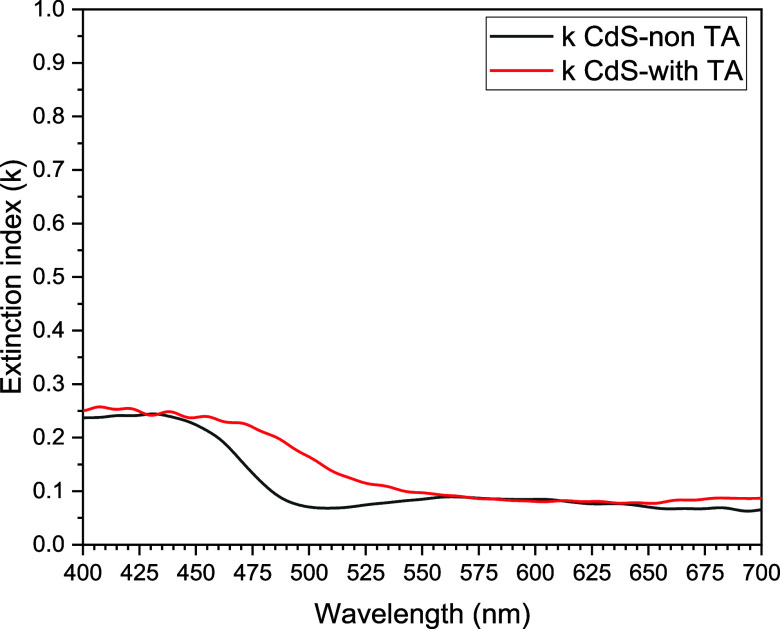
Extinction coefficient comparison between CdS TFs without thermal annealing (black) and CdS TFs with thermal annealing (red). There is a displacement among them.

The extinction coefficients of both CdS layers considered above were calculated using the expression shown in eq 3. As can be seen, the extinction coefficients are small, which we associate as an index of transparency or low dispersion of electromagnetic waves in the visible region. In addition, as the previously analyzed optical properties, presently, a characteristic step behavior of electronic transitions of the considered CdS semiconductor films is shown, see Figure 5.
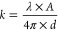
3

**Figure 6:**
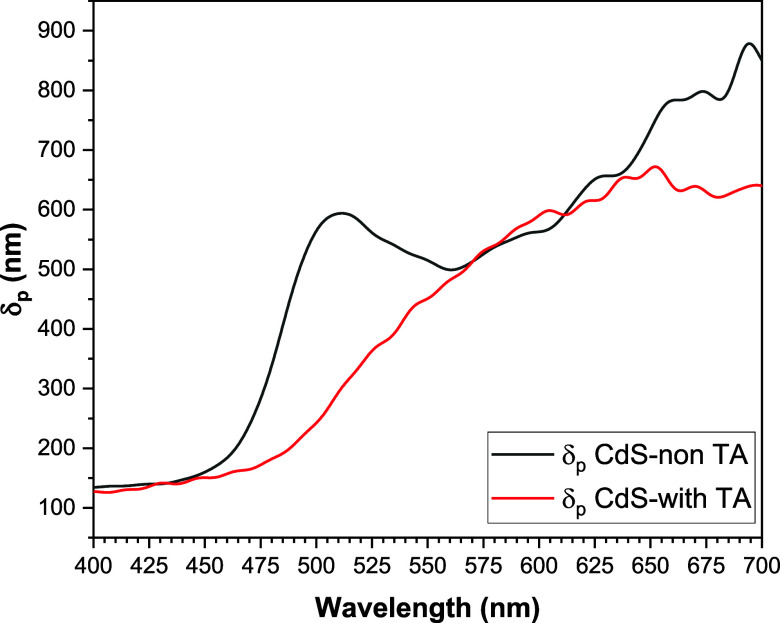
Deep light penetration for an  intensity attenuation of CdS TFs without and with thermal annealing, black and red, respectively.

From the optical absorptions (*A*) of our materials and their thickness, it is possible to obtain their absorbance (alpha) as well as an estimation of the depth of penetration of the electromagnetic waves in the visible region, see Figure 6, for an attenuation of intensities of 1/e. The mathematical relationships that support this procedure correspond to eqs 4, 5 and 6. In this analysis, it is determined that the CdS film without thermal annealing allows a greater penetration of light in the region from 445 nm until 570 nm.
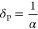
4
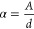
5
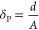
6

**Figure 7:**
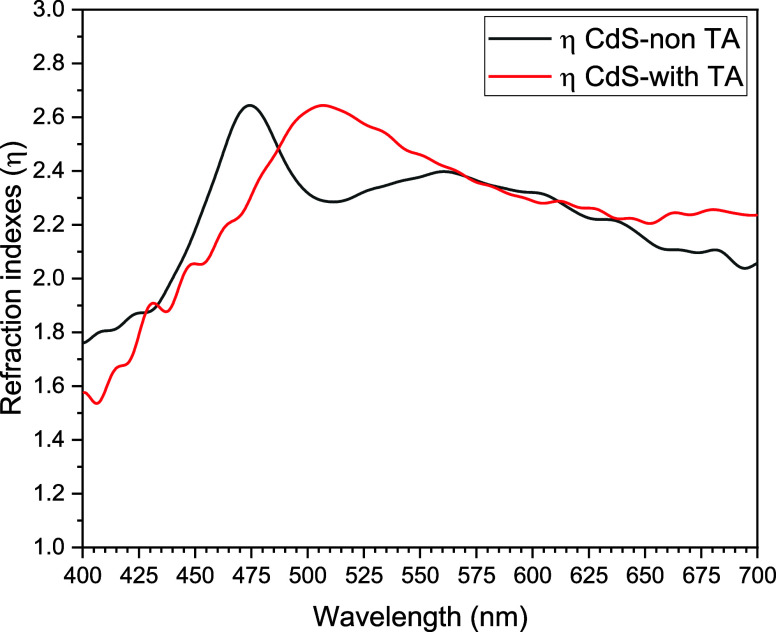
Plots of refraction indexes of CdS TFs at the ground state (black) and CdS TFs with thermal annealing at 250 °C for 5 min (red).

There is a relation between the refraction index (η), extinction coefficient (*k*), and reflection (*R*) useful to calculate the refraction index properties for our CdS TFs; these mathematical expressions are denoted in eqs 7 and 8. Figure 7 shows the comparison of these indexes for our CdS TFs; here, the maximum value for CdS without thermal annealing is located at 474 nm, while that for CdS with thermal annealing is located at 507 nm.
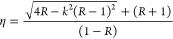
7

If *k* < 1, then *k*^2^ ≪ 1, so
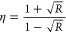
8

Direct bandgap obtained for the considered CdS TFs was determined and justified by the Tauc procedure, which establishes the square of the product of the optical absorption times the energy of the incident photons that interact with a material; after a certain stability, it reaches a linear behavior with such an energy that is receiving, which is representative of its electronic transitions.

This is represented in the expressions of eqs 9 and 10.
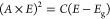
9

For (*A* × *E*)^2^ = 0
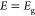
10

**Figure 8:**
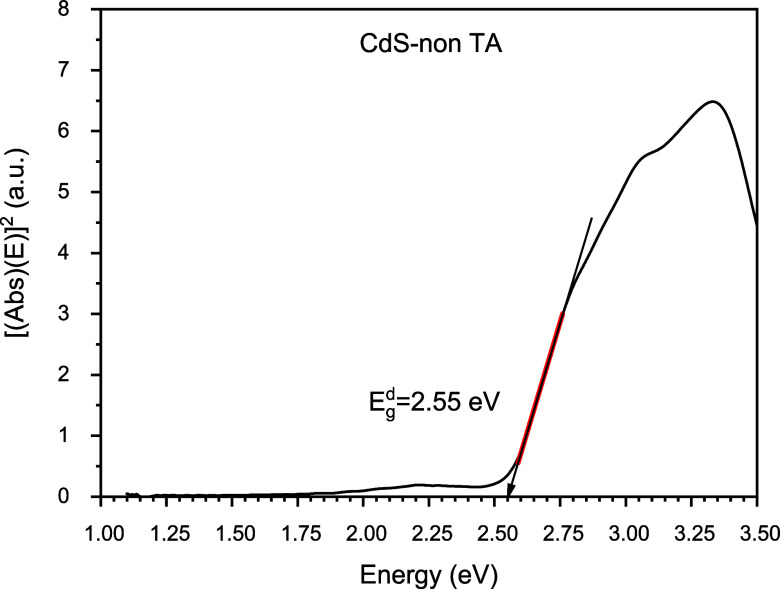
Graphic representation of the bandgap calculation for CdS without thermal annealing.

Figure 8 corresponds to the direct bandgap for CdS TFs without thermal annealing, giving a value of 2.55 eV in complete concordance with the reported data.

**Figure 9:**
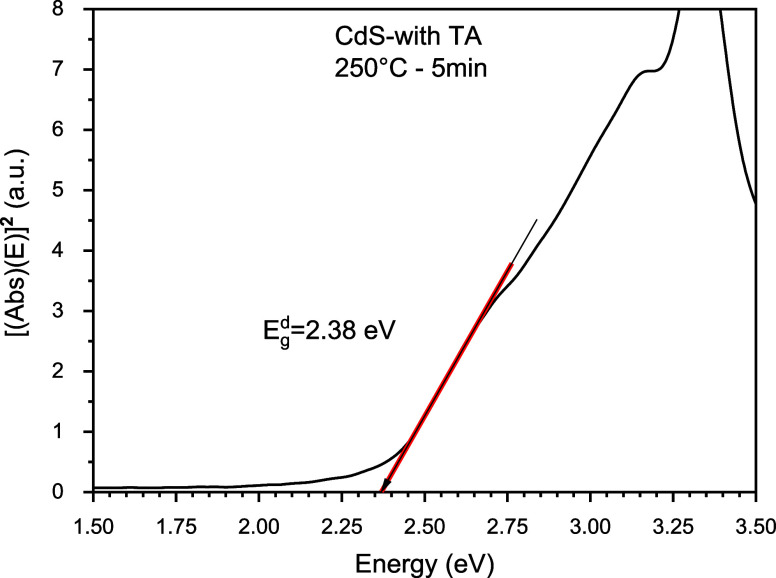
Graphic representation of bandgap calculation for CdS with thermal annealing at 250 °C for 5 min.

On the other hand, we proceed to calculate the direct bandgap of a CdS TF with thermal annealing at 250 °C for 5 min. A bandgap of 2.38 (eV) was obtained, which is lower than that of the CdS TF as grown. This can be observed in Figure 9.

**Figure 10:**
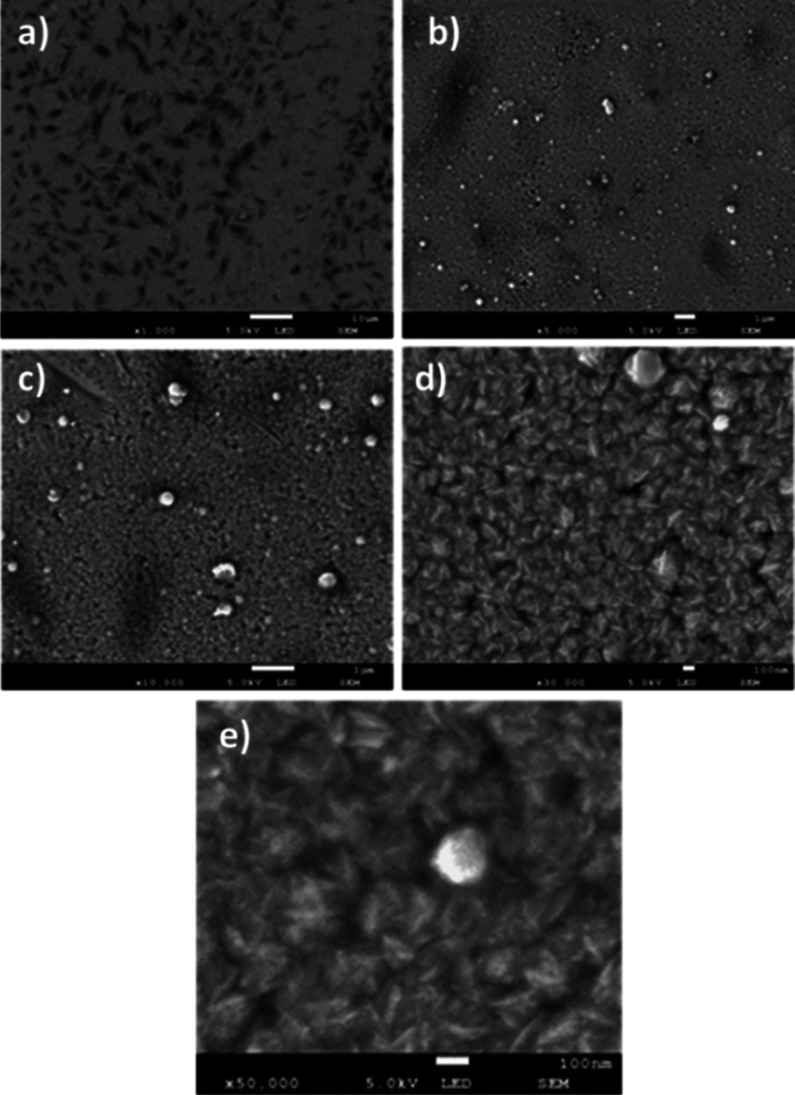
Different magnifications of the morphology of the CdS TF without thermal annealing: (a) 1000×, (b) 5000×, (c) 10,000×, (d) 30,000×, and (e) 50×000×.

The following characterization corresponds to the morphology of the n-type material, CdS, without subjecting it to thermal annealing. Figure 10 shows 5 images, (a), (b), (c), (d), and (e), which correspond to magnifications of 1000×, 5000×, 10,000×, 30,000×, and 50,000×, respectively. In the first scale, randomly oriented elongated formations are observed; in the next magnification, a rounded granular morphology of CdS clusters was slightly observed, of several sizes between 100 and 600 nm, with the appearance of an apparently flat bottom. The following magnification shows the CdS clusters with a rough morphology. The last two magnifications show a better sharpness or resolution of the morphology at the nanostructured scale.

**Figure 11:**
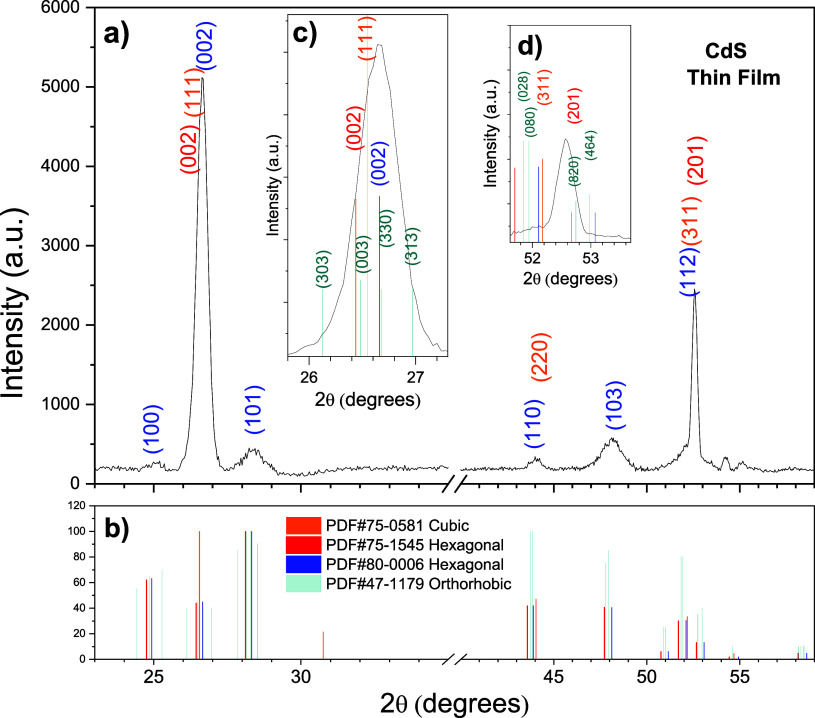
CdS XRD on the glass substrate, showing (a) complete pattern and (b) three PDFs matching with the experimental pattern. (c,d) Insets remark different phases of CdS in the two most intense peaks.

Figure 11 shows an experimental X-ray diffraction (XRD) pattern on a glass substrate for a CdS TF. As can be observed, Figure 11a shows a complete pattern, and Figure 11b shows three PDFs matching with the experimental pattern. The inset figures, Figure 11c,d, show the different compositions of CdS in the two most intense peaks.

**Figure 12:**
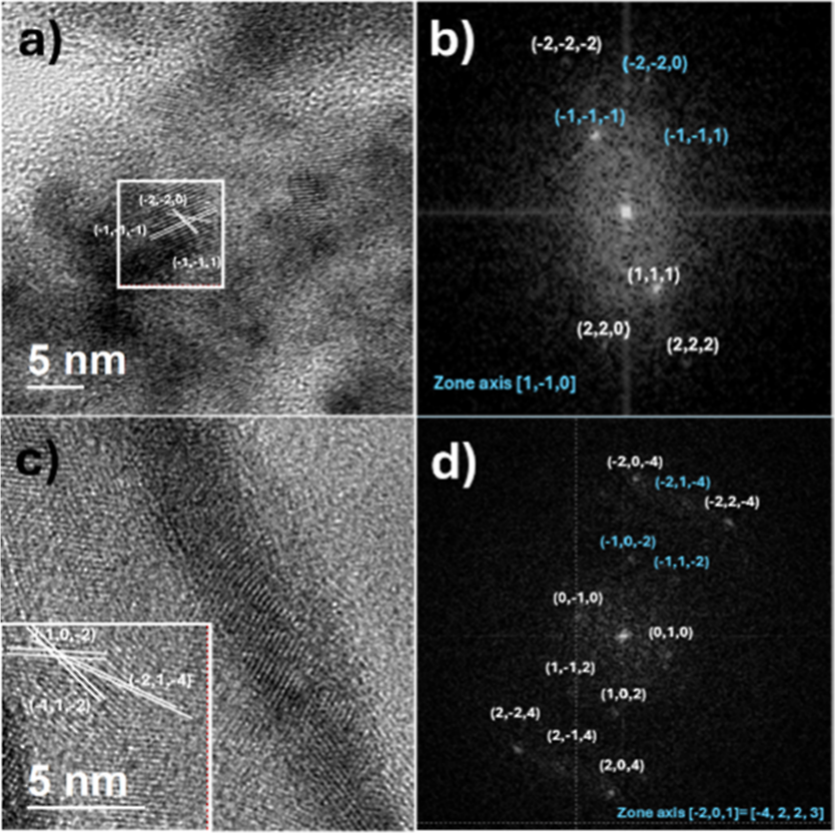
(a) shows the TEM image of powder detached from the CdS TF, (b) shows its fast Fourier transform, some Miller indexes had been located, and (c,d) are for other portion or micrograph of the sample.

Powder of CdS was detached from a CdS TF in order to be observed by high-resolution transmission electron microscopy (HRTEM). Figure 12 depicts the achieved information obtained, which matches with a cubic structure PDF#75–0581, into a region of the powder parts (a) and (b) of Figure 12, while the other region of the powder parts matches with a hexagonal structure PDF#80–0006, parts (c) and (d) of Figure 12.

**Figure 13:**
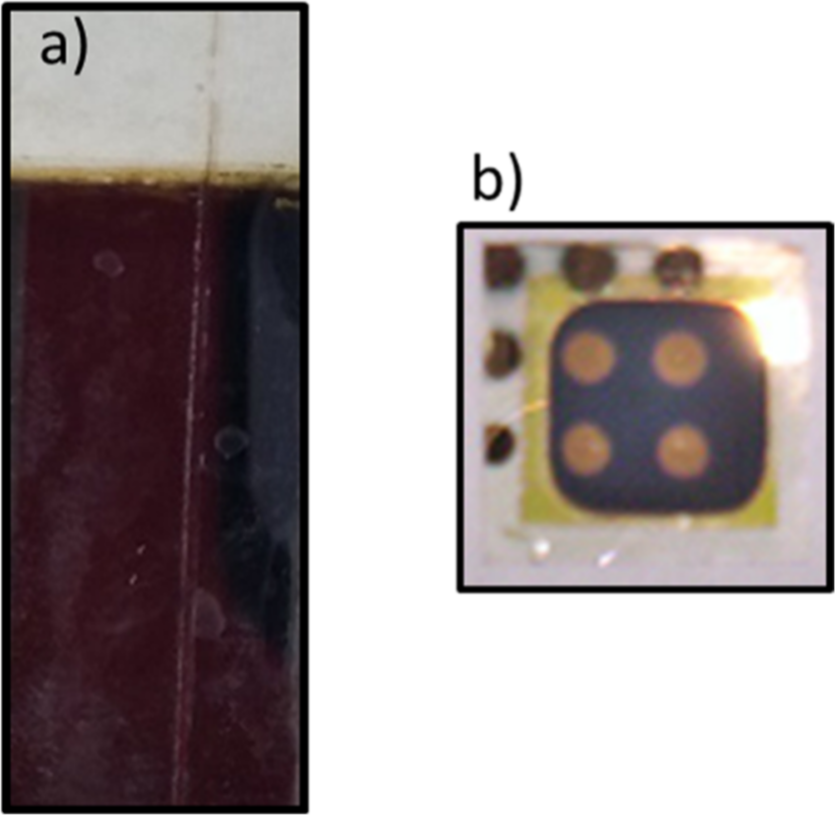
Photographs corresponding to (a) PbS/sodalime-glass and (b) diodic bilayer PbS/CdS/sodalime-glass on the base of the thermally annealed system.

Now, we will describe the characterizations carried out on the second semiconductor TF of PbS; Figure 13a shows the deposition of PbS on a sodalime-type glass substrate, with the appearance of a brownish dark color. Figure 13b shows the deposition of PbS on the CdS film previously deposited on a glass substrate coated with ITO; a contact distribution can also be seen to perform a fundamental electrical evaluation. The CdS/ITO/glass-chosen system was thermally annealed.

The PbS TF that grew to form the planned device was deposited on a sodalime glass substrate, and after being separated for observation by TEM, it gave a cubic crystal structure, corresponding to a pattern in the database, PDF#05–0592.

**Figure 14:**
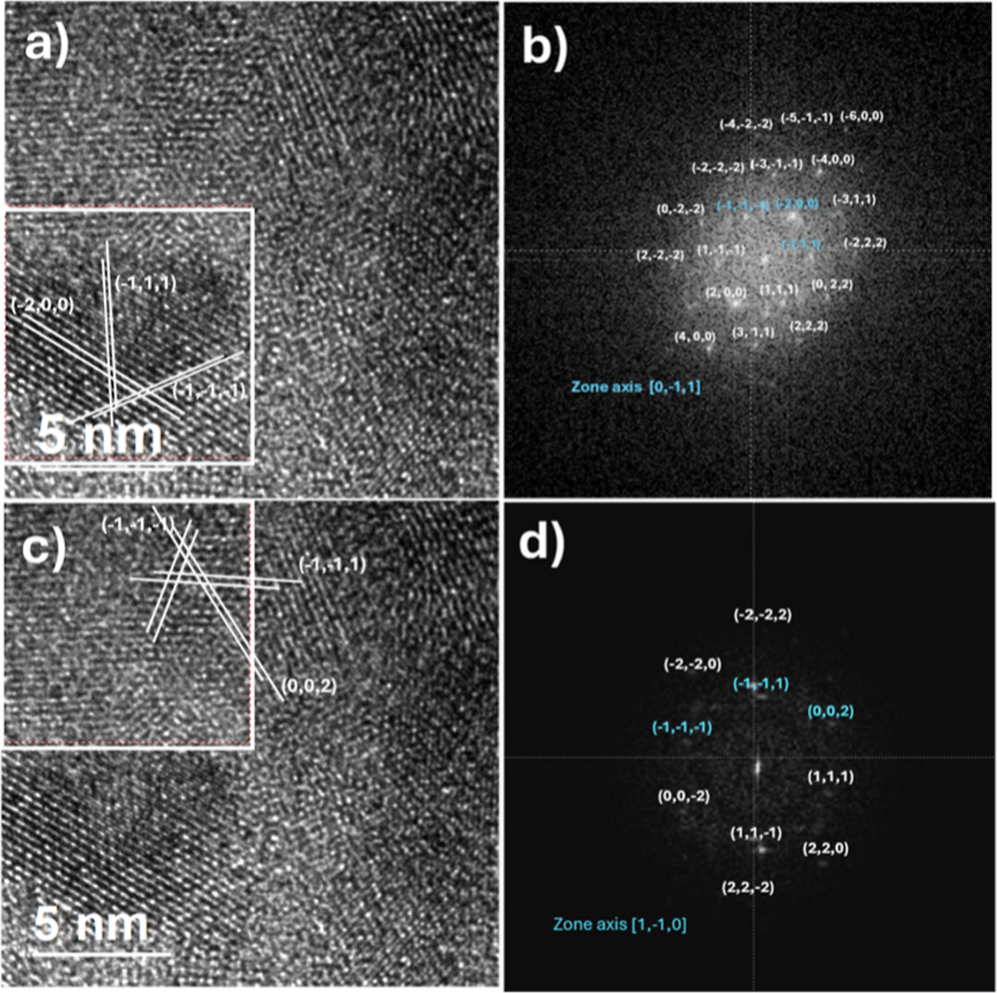
(a,c) Two representative zones into the same PbS sample and (b,d) their fast Fourier transform.

Figure 14 shows a pair of zones into a micrograph of a PbS sample for the device, and their corresponding fast Fourier transforms are shown in Figure 14(a,c) and (b,d), respectively.

**Figure 15:**
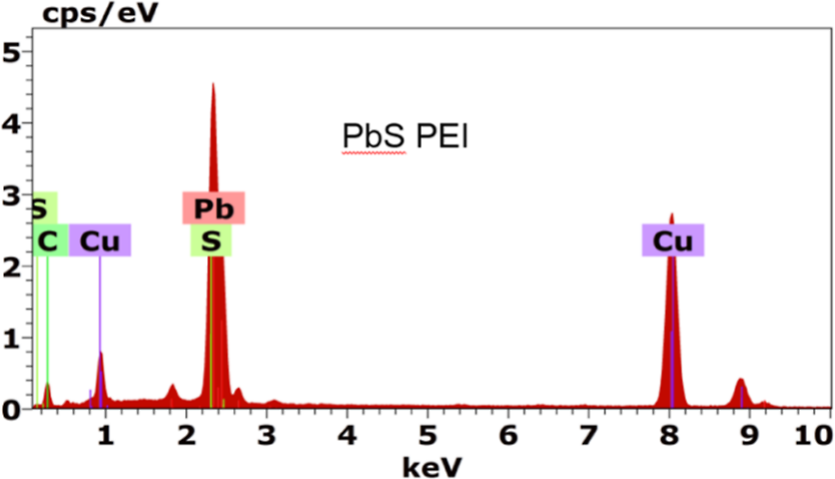
Distribution of elements that are present in a characteristic sample of the PbS TF synthesized in our diode.

Our transmission electron microscope is equipped with the energy-dispersive spectroscopy technique, so that we can measure the chemical composition of a PbS characteristic sample, in order to verify its present elements. Figure 15 shows the presence of Pb, S, C, and Cu; the carbon and copper peaks are due to the sample holder composition. Then, the remaining elements are due to our considered material.

**Figure 16:**
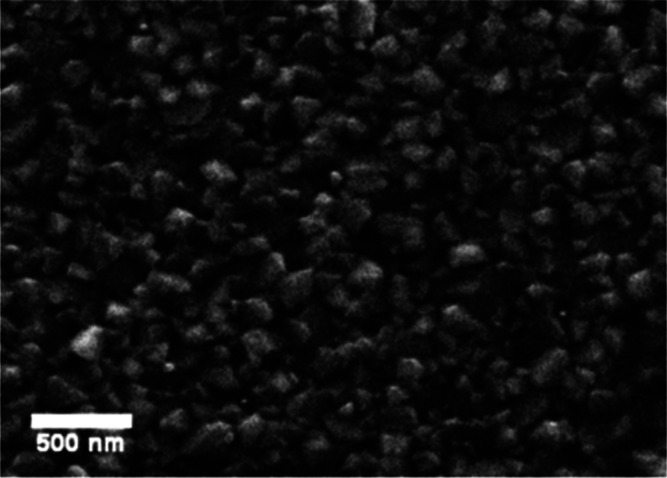
Characteristic SEM images taken from PbS TFs, showing the formation of a granular morphology of similar sizes and of angular shapes.

Figure 16 shows the scanning electron microscopy (SEM) surface morphology of a PbS TF deposited on a glass substrate, which can be served as a surface formed by angular grains, distributed homogeneously.

This morphology is slightly different from that typically reported in scientific literature, and it is due to the used complexing agent.

**Figure 17:**
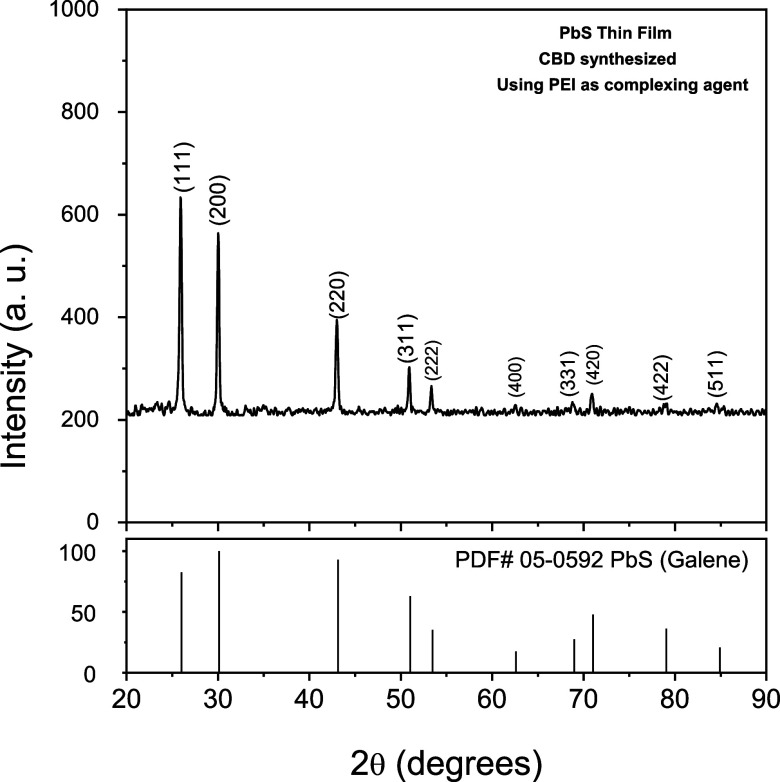
XRD pattern, corresponding to the synthesized PbS material in this investigation, for the elaboration of a photosensitive diode.

On the other hand, the PbS material was also characterized structurally by using the XRD technique, where the identity of such a material was established, showing a simple cubic structure, corresponding to the PDF# 05–0592 pattern. Figure 17 shows the diffraction pattern obtained compared to a reference diffraction pattern in the database.

**Figure 18:**
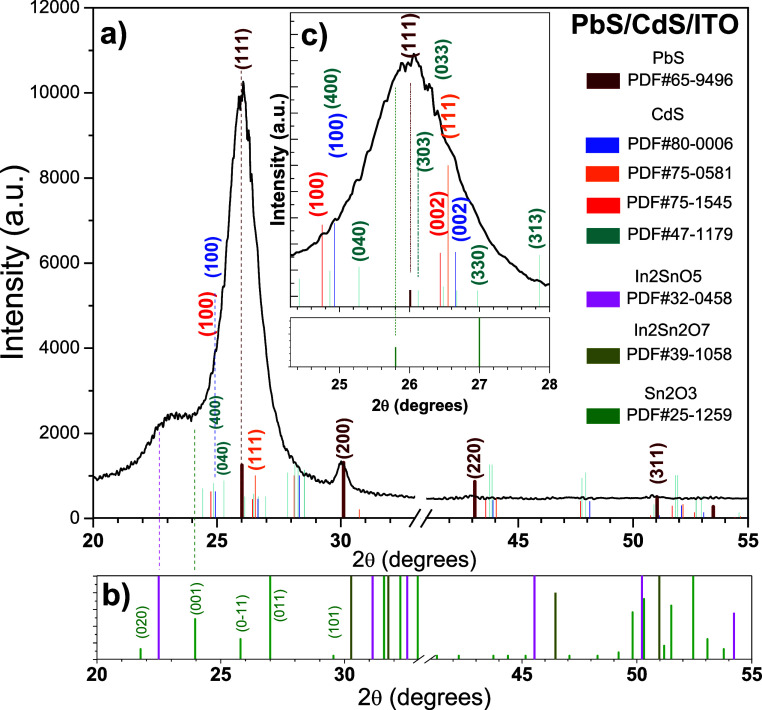
XRD pattern, corresponding to the system PbS/CdS/ITO.

Figure 18 shows a corresponding pattern of the complete multilayer device. As can be observed, there are peaks of cubic PbS; hexagonal, cubic, and orthorhombic CdS phases; and some additional peaks of In_2_SnO_5_, In_2_Sn_2_O_7_, and Sn_2_O_3_.

In the context of the discussion, it can be added that there are some similar works in the scientific literature; however, our contribution is to provide a straightforward methodology for the fabrication of the diodic bilayer. Furthermore, our research group is the pioneer of the formulation used for the PbS TF.^76,77,84,88^

**Figure 19:**
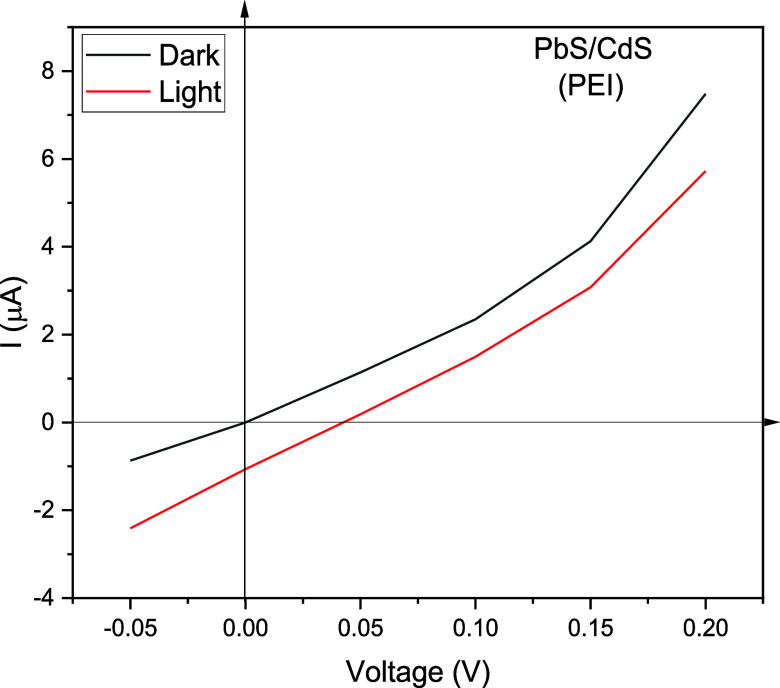
*I*–*V* characteristic curves for the PbS/CdS diode; the black line corresponds to the dark condition and the red line to the lighting condition.

Finally, in Figure 19, we show a very fundamental electrical characterization of the PbS/CdS bilayer system, in which metallic electrodes are placed in contact with the PbS and the ITO electrode is in contact with CdS, generating the *I*–*V* curves from −0.05 to 0.20 V. Two corresponding characteristic curves with two lighting conditions were obtained, darkness and a controlled fixed lighting. As observed, the behavior of the PbS/CdS bilayer moved due to the effect of lighting, thus demonstrating photosensitivity.

The photosensitive diode was stimulated by an incident power *P*_0_ = 9.37488 × 10^–4^*w* considering as response area the circles of metallic contacts placed on the PbS film with a diameter of 3 mm (*A* = 7.0685 × 10^–6^*m*^2^) and using a 75w lamp with a reflective screen placed at a distance of 30 cm from the diode. The calculation of the maximum electrical power was estimated for the values *I* = 0.6966 × 10^–6^*A*, *V* = 0.0152872*V* I, obtaining an electrical power of *P*_*e*_ = 1.0683 × 10^−8^*w*.

## 4 Conclusions

The MH, Ag/PbS/CdS/ITO/glass, was fabricated successfully by the CBD technique in a 1×1 in. area.

Both CdS (yellow color) and PbS (black color) TFs are homogeneous and present good adhesion to the substrate.

In Figure 4, a significant change in absorption edge is observed, decreasing the bandgap from 2.55 to 2.38 eV, see Figures 8 and 9.

From Figure 6, it is concluded that thermal annealing of the CdS film considerably improved the depth of light penetration, in the range of 450–550 nm, making it a better optical window material.

From the X-ray diffractograms, it is verified that the crystalline structure of the CdS film presents a mixture of two phases, hexagonal and cubic, being mostly hexagonal, while the diffractogram of the PbS film shows a cubic structure.

Finally, from the *I* versus *V* measurements, the photovoltaic effect and a slightly diodic behavior in dark conditions are verified.

## References

[ref1] Rahman F. Zinc oxide light-emitting diodes: a review. Opt. Eng. 2019, 58 (01), 010901 10.1117/1.OE.58.1.010901.

[ref2] Gupta S. K.; Modak B.; Abraham M.; Das S.; Gupta R.; Girija K. G.; Mohapatra M.; Sudarshan K. Defect induced tunable light emitting diodes of compositionally modulated zinc gallium germanium oxides. Chem. Eng. J. 2023, 474, 145595 10.1016/j.cej.2023.145595.

[ref3] Das H. S.; Mishra S.; Dash M. K.; Nandi P. K.; Maity S. K.; Khatua D.; Chatterjee A.; Guo z.; Xu B. B.; Roymahapatra G. Transparent Conducting Gallium-Doped Zinc Oxide Thin Films on Glass Substrate for Optoelectronic Device Applications. ES Mater. Manuf. 2023, 22, 841 10.30919/esmm5f841.

[ref4] Shanivarasanthe Nithyananda Kumar R.; Breugelmans R.; Jiang X.; Ahadzadeh S.; Brammertz G.; Verding P.; Daenen M.; Van Landeghem M.; Cambré S.; Vandewal K.; Deferme W. Organic- inorganic nanoparticle composite as an electron injection/hole blocking layer in organic light emitting diodes for large area lighting applications. Appl. Surf. Sci. 2023, 631, 157548 10.1016/j.apsusc.2023.157548.

[ref5] Chakraborty M.; Banerjee D.; Singh S.; Dutta J. Photoluminescence and EPR investigation in ZnO: Gd3+, Yb3+ phosphors for application in light emitting diode. Mater. Sci. Semicond. Process. 2023, 166, 107758 10.1016/j.mssp.2023.107758.

[ref6] Wang H.; Tang X.; Sun G.; Chen D.; Ye Z.; Liu Y.; Jin Y. All-Solution-Processed Perovskite Light-Emitting Diodes Based on Thiol-Modified ZnO Electron-Transporting Layer. J. Phys. Chem. Lett. 2023, 14, 5827–5883. 10.1021/acs.jpclett.3c01428. 37339376

[ref7] Du Y.; Gao Y.; Si J.; Du Z.; Xu R.; Hu Q.; Hao X.; Gong X.; Zhang Z.; Zhao H.; Cai P.; Ai Q.; Yao X.; Cai M.; Ye Z.; Dai X.; Liu Z. In-Situ Interfacial Reaction Induced Amino-Rich Oxide Surface to Grow High-Quality FAPbBr3 Crystals for Efficient Inverted Light-Emitting Diodes. ACS Mater. Lett. 2023, 5 (4), 1179–1187. 10.1021/acsmaterialslett.3c00039.

[ref8] Nagata K.; Matsubara T.; Saito Y.; Kataoka K.; Narita T.; Horibuchi K.; Kushimoto M.; Tomai S.; Katsumata S.; Honda Y.; Takeuchi T.; Amano H. A Review on the Progress of AlGaN Tunnel Homojunction Deep-Ultraviolet Light-Emitting Diodes. Crystals 2023, 13 (3), 524 10.3390/cryst13030524.

[ref9] Lopez-Martin A. J.; Carlosena A. Contactless potentiometers for automotive applications. 2013 Seventh International Conference on Sensing Technology (ICST); IEEE, 2013; pp 361–364.

[ref10] Li B.; Wang F.; Jiang F.; Zhao S.; Wei S.; Peng P.; Wang X.; Jiang A. Performance Study of Portable Semiconductor Refrigeration Device Based on CFD Simulation. Micromachines 2023, 14 (2), 296 10.3390/mi14020296. 36837996 PMC9961615

[ref11] Rim Y. S. Review of metal oxide semiconductors-based thin-film transistors for point-of-care sensor applications. J. Inf. Disp. 2020, 21 (4), 203–210. 10.1080/15980316.2020.1714762.

[ref12] Panca A.; Panidi J.; Faber H.; Stathopoulos S.; Anthopoulos T. D.; Prodromaki T. Review Flexible Oxide Thin Film Transistors, Memristors, and Their Integration. Adv. Funct. Mater. 2023, 33, 2213762 10.1002/adfm.202213762.

[ref13] Lim S. H.; Kim T. I.; Park I. J.; Kwon H. I. Synthesis of a Tellurium Semiconductor with an Organic–Inorganic Hybrid Passivation Layer for High-Performance p Type Thin Film Transistors. ACS Appl. Electron. Mater. 2023, 5 (9), 4816–4825. 10.1021/acsaelm.3c00620.

[ref14] Yap B. K.; Zhang Z.; Thien G. S. H.; Chan K. Y.; Tan C. Y. Recent advances of In2O3-based thin-film transistors: A review. Appl. Surf. Sci. Adv. 2023, 16, 100423 10.1016/j.apsadv.2023.100423.

[ref15] Jeon Y.; Lee D.; Yoo H. Review Recent Advances in Metal-Oxide Thin-Film Transistors: Flexible/Stretchable Devices. Integrated Circuits, Biosensors, and Neuromorphic Applications. Coatings 2022, 12, 204 10.3390/coatings12020204.

[ref16] Shi R.; Lei T.; Xia Z.; Wong M. Low-temperature metal–oxide thin-film transistor technologies for implementing flexible electronic circuits and systems. J. Semicond. 2023, 44 (9), 091601 10.1088/1674-4926/44/9/091601.

[ref17] Chen F.; Zhang M.; Wan Y.; Xu X.; Wong M.; Kwok H. S. Advances in mobility enhancement of ITZO thin-film transistors: a review. J. Semicond. 2023, 44 (9), 091602 10.1088/1674-4926/44/9/091602.

[ref18] Lin D.; Yang J. Z.; Cheng J. R.; Deng X. C.; Chen Y. S.; Zhuang P. P.; Li T. J.; Liu J. InSnO:N homojunction thin-film transistors fabricated at room temperature. Vacuum 2023, 213, 112099 10.1016/j.vacuum.2023.112099.

[ref19] Kumar A.; Goyal A. K.; Gupta N. Review—Thin-Film Transistors (TFTs) for Highly Sensitive Biosensing Applications: A Review. ECS J. Solid State Sci. Technol. 2020, 9 (11), 115022 10.1149/2162-8777/abb2b3.

[ref20] Jenifer K.; Arulkumar S.; Parthiban S.; Kwon J. Y. A Review on the Recent Advancements in Tin Oxide-Based Thin-Film Transistors for Large-Area Electronics. J. Electron. Mater. 2020, 49 (12), 7098–7111. 10.1007/s11664-020-08531-x.

[ref21] Ji D.; Jang J.; Park J. H.; Kim D.; Rim Y. S.; Hwang Do K.; Noh Y.-Y. Progress Report Recent progress in the development of backplane thin film transistors for information displays. J. Inf. Disp. 2021, 1 (22), 1–11. 10.1080/15980316.2020.1818641.

[ref22] Zhang L.; Yu H.; Xiao W.; Liu C.; Chen J.; Guo M.; Gao H.; Liu B.; Wu W. Strategies for Applications of Oxide-Based Thin Film Transistors. Electronics 2022, 11 (6), 960 10.3390/electronics11060960.

[ref23] Lee T. D.; Ebong A. U. A review of thin film solar cell technologies and challenges. Renew. Sustain. Energy Rev. 2017, 70, 1286–1297. 10.1016/j.rser.2016.12.028.

[ref24] Sivaraj S.; Rathanasamy R.; Kaliyannan G. V.; Panchal H.; Jawad Alrubaie A.; Musa Jaber M.; Said Z.; Memon S. A Comprehensive Review on Current Performance, Challenges and Progress in Thin-Film Solar Cells. Energies 2022, 15 (22), 8688 10.3390/en15228688.

[ref25] Soonmin H.; Hardani; Nandi P.; Mwankemwa B. S.; Malevu T. D.; Malik M. I. Overview on Different Types of Solar Cells: An Update. Appl. Sci. 2023, 13 (4), 2051 10.3390/app13042051.

[ref26] Ahmad K. S.; Naqvi S. N.; Jaffri S. B. Review article Systematic review elucidating the generations and classifications of solar cells contributing towards environmental sustainability integration. Rev. Inorg. Chem. 2021, 41 (1), 21–39. 10.1515/revic-2020-0009.

[ref27] Soonmin H. A Review of Metal Oxide Thin Films in Solar Cell Applications. Int. J. Thin Films Sci. Technol. 2022, 11 (1), 37–45. 10.18576/ijtfst/110105.

[ref28] Thin Film Phenomena; Chopra K. L., Ed.; McGraw-Hill, Inc: New York, 1969.

[ref29] Handbook of Thin Film Technology; Maissel L. I., Glang R., Eds.; McGraw-Hill, Inc: New York, 1970.

[ref30] Handbook of Thin-Film Deposition Processes and Technique Principles, Methods, Equipment and Applications, 2nd ed.; Seshan K., Ed.; Noyes Publications: New York, 2002.

[ref31] Poonam A study on the thin films deposition technique. Int. J. Eng. Sci. Math. 2017, 4 (6), 175–181.

[ref32] Springer Handbook of Electronic and Photonic Materials, 2nd ed.; Kasap S., Capper P., Eds.; Springer, 2017.

[ref33] Oluwatosin Abegunde O.; Titilayo Akinlabi E.; Philip Oladijo O.; Akinlabi S.; Uchenna Ude A. Overview of thin film deposition techniques. Mater. Sci. 2019, 6 (2), 174–199. 10.3934/matersci.2019.2.174.

[ref34] Geremew T. Thin Film Deposition and Characterization Techniques. J. 3D Print. Appl. 2022, 1 (2), 1–24. 10.14302/issn.2831-8846.j3dpa-22-4066.

[ref35] Thin Film Structures in Energy Applications; Moorthy S. B. K., Ed.; Springer, 2015.

[ref36] Physics of Thin Films; Eckertová L., Ed.; Springer Science & Business Media: New York, 1977.

[ref37] Engineering Materials Contemporary Nanomaterials in Material Engineering Applications; Mubarak N. M., Khalid M., Walvekar R., Numan A., Eds.; Springer, 2021.

[ref38] Chemical Solution Deposition of Semiconductor Films; Hodes G., Ed.; Marcel Dekker, Inc: New York, 2002.

[ref39] Chemical Bath Deposition. In Chemical Solution Deposition of Functional Oxide Thin Films; Schneller T.; Waser R., Kosec M.; Payne D., Eds.; Springer, 2013; Chapter 14.

[ref40] Niesen T. P.; De Guire M. R. Review: Deposition of Ceramic Thin Films at Low Temperatures from Aqueous Solutions. J. Electroceram. 2001, 6 (3), 169–207. 10.1023/a:1011496429540.

[ref41] Ezekoye B. A.; Offor P. O.; Ezekoye V. A.; Ezema F. I. Chemical Bath Deposition Technique of Thin Films: A Review. Int. J. Sci. Res. 2013, 2 (8), 452–456. 10.15373/22778179/aug2013/149.

[ref42] Mane R. S.; Lokhande C. D. Chemical deposition method for metal chalcogenide thin films. Mater. Chem. Phys. 2000, 65 (1), 1–31. 10.1016/S0254-0584(00)00217-0.

[ref43] Grozdanov I. A simple and low-cost technique for electroless deposition of chalcogenide thin films. Sci. Technol. 1994, 9 (6), 1234–1241. 10.1088/0268-1242/9/6/013.

[ref44] Hone F. G.; Abza T. Short Review of Factors Affecting Chemical Bath Deposition Method for Metal Chalcogenide thin films. Int. J. Thin Films Sci. Technol. 2019, 8 (2), 43–53. 10.18576/ijtfst/080203.

[ref45] Pawar S. M.; Pawar B. S.; Kim J. H.; Joo Oh-S.; Lokhande C. D. Recent status of chemical bath deposited metal chalcogenide and metal oxide thin films. Curr. Appl. Phys. 2011, 11 (2), 117–161. 10.1016/j.cap.2010.07.007.

[ref46] Sengupta S.; Aggarwal R.; Raula M. A review of chemical bath deposition of metal chalcogenide thin films for heterojunction solar cells. J. Mater. Res. 2023, 38, 142 10.1557/s43578-022-00539-9.

[ref47] Soonmin H.; Pronoy N.; Nandi P.; Mwankemwa B. S.; Malevu T. D.; Malik M. I. Overview on Different Types of Solar Cells: An Update. Appl. Sci. 2023, 13 (4), 2051 10.3390/app13042051.

[ref48] Sivaraj S.; Rathanasamy R.; Kaliyannan G. V.; Panchal H.; Jawad Alrubaie A.; Musa Jaber M.; Said Z.; Memon S. A Comprehensive Review on Current Performance, Challenges and Progress in Thin-Film Solar Cells. Energies 2022, 15 (22), 8688 10.3390/en15228688.

[ref49] Ahmad K. S.; Naqvi S. N.; Jaffri S. B. Systematic review elucidating the generations and classifications of solar cells contributing towards environmental sustainability integration. Inorg. Chem. 2021, 41 (1), 21–39. 10.1515/revic-2020-0009.

[ref50] Lee T. D.; Ebong A. U. A review of thin film solar cell technologies and challenges. Energy Rev. 2017, 70, 1286–1297. 10.1016/j.rser.2016.12.028.

[ref51] Rafiee M.; Chandra S.; Ahmed H.; McCormack S. J. An overview of various configurations of Luminescent Solar Concentrators for photovoltaic applications. Opt. Mater. 2019, 91, 212–227. 10.1016/j.optmat.2019.01.007.

[ref52] Ahmad F.; Qurban N.; Fatima Z.; Ahmad T.; Zahid I.; Ali A.; Rajpoot S. R.; Tasleem M. W.; Maqbool E. Electrical Characterization of II-VI Thin Films for Solar Cells Application. J. Sci. Eng. 2022, 2 (3), 199–208. 10.17509/ajse.v2i3.39425.

[ref53] Machol J. L.; Wise F. W.; Patel R. C.; Tanner D. B. Vibronic quantum beats in PbS microcrystallites. Phys. Rev. B 1993, 48 (4), 2819–2822. 10.1103/physrevb.48.2819. 10008690

[ref54] Schmitt-Rink S.; Miller D. A. B.; Chemla D. S. Theory of the linear and nonlinear optical properties of semiconductor microcrystallites. Phys. Rev. B 1987, 35 (15), 8113–8125. 10.1103/physrevb.35.8113. 9941148

[ref55] Scanlon W. W.; Scanlon Intrinsic Optical Absorption and the Radiative Recombination Lifetime in PbS. Phys. Rev. 1958, 109 (1), 47–50. 10.1103/physrev.109.47.

[ref56] Ezenwa I. A. Short Communication Effect of Film Thickness on the Transmittivity of Chemical Bath Synthesized PbS Thin Film Research. J. Eng. Sci. 2013, 2 (2), 23–25.

[ref57] Ezekoye B. A.; Emeakaroha T. M.; Ezekoye V. A.; Ighodalo K. O.; Offor P. O. Optical and structural properties of lead sulphide (PbS) thin films synthesized by chemical method. Int. J. Phys. Sci. 2015, 10 (13), 385–390. 10.5897/IJPS2015.4354.

[ref58] Batu B.; Tamasgen T. Effects of Thickness on Optical and Structural Properties of Lead Sulphide (PbS) Thin Film Prepared by Chemical Bath Deposition (CBD). Chem. Mater. Res. 2020, 12, 7 10.7176/CMR/12-7-01.

[ref59] Mosiori O. C.; Njoroge W. N.; Okumu J. Optical and Electrical Properties of Pbs Thin Films Grown by Chemically Bath Deposition [CBD] at Different Lead Concentrations. Int. J. Adv. Res. Phys. Sci. 2014, 1 (1), 25–32.

[ref60] Maharnavar B. S.; Bagal M. G.; Jadhav N. A.; Pardeshi A. R.; Pingale P. C. Synthesis of PbS Thin Film by Chemical Bath Deposition Method and it’s structural-Optical Studies. J. Emerg. Technol. Innovat. Res. 2021, 8 (3), 2776–2781.

[ref61] Rohom A. B.; Londhe P. U.; Jadhav P. R.; Bhand G. R.; Chaure N. B. Studies on chemically synthesized PbS thin films for IR detector application. J. Mater. Sci.: Mater. Electron. 2017, 28 (22), 17107–17113. 10.1007/s10854-017-7637-4.

[ref62] Pop I.; Nascu C.; Ionescu V.; Indrea E.; Bratu I. Structural and optical properties of PbS thin films obtained by chemical deposition. Thin Solid Films 1997, 307 (1–2), 240–244. 10.1016/S0040-6090(97)00304-0.

[ref63] Thangaraju B.; Kaliannan P. Spray pyrolytically deposited PbS thin films. Semicond. Sci. Technol. 2000, 15 (8), 849–853. 10.1088/0268-1242/15/8/311.

[ref64] Xu W.; Tan F.; Liu Q.; Liu X.; Jiang Q.; Wei L.; Zhang W.; Wang Z.; Qu S.; Wang Z. Efficient PbS QD solar cell with an inverted structure. Sol. Energy Mater. Sol. Cells 2017, 159, 503–509. 10.1016/j.solmat.2016.10.006.

[ref65] Lilhare D.; Khare A. Review Development of chalcogenide solar cells: Importance of CdS window layer. Opto-Electron. Rev. 2020, 28, 43–63. 10.24425/opelre.2020.132500.

[ref66] Najm A. S.; Naeem H. S.; Majdi H. Sh.; Hasbullah S.A.; Hasan H.A.; Sopian K.; Bais B.; Al Iessa H. J.; Dhahad H.A.; Ali J. M.; Sultan A. J. An in-depth analysis of nucleation and growth mechanism of CdS thin film synthesized by chemical bath deposition (CBD) technique. Sci. Rep. 2022, 12 (1), 15295 10.1038/s41598-022-19340-z. 36096904 PMC9468032

[ref67] Moreno-Regino V. D.; Castañeda-de-la-Hoya F.; Torres-Castanedo C.; Márquez-Marín J.; Castanedo-Pérez R.; Torres-Delgado G.; Zelaya-Ángel O. Structural, optical, electrical and morphological properties of CdS films deposited by CBD varying the complexing agent concentration. Results Phys. 2019, 13 (13), 102238 10.1016/j.rinp.2019.102238.

[ref68] Subba Ramaiah K.; Pilkington R. D.; Hill A. E.; Tomlinson R. D.; Bhatnagar A. K. Structural and optical investigations on CdS thin films grown by chemical bath technique. Mater. Chem. Phys. 2001, 68 (1–3), 22–30. 10.1016/s0254-0584(00)00281-9.

[ref69] Ashok A.; Regmi G.; Romero-Núñez A.; Solis-López M.; Velumani S.; Castaneda H. Comparative studies of CdS thin films by chemical bath deposition techniques as a buffer layer for solar cell applications. J. Mater. Sci. Mater. Electron. 2020, 31 (10), 7499–7518. 10.1007/s10854-020-03024-3.

[ref70] Salim H. I. The effect of growth technique on the characteristic properties of CdS layers for solar cell applications. J. Mater. Sci.: Mater. Electron. 2020, 31 (5), 4193–4207. 10.1007/s10854-020-02972-0.

[ref71] Ortuño-López M. B.; Ochoa-Landín R.; Sandoval-Paz M. G.; Sotelo-Lerma M.; Flores-Acosta M.; Ramírez-Bon R. Studies on the Properties of CdS Films Deposited from pH-controlled Growth Solutions. Mater. Res. 2013, 16, 937–943. 10.1590/S1516-14392013005000103.

[ref72] Vázquez-Monroy F.; García-Barrientos A.; Hoyo-Montanño J. A.; Valencia-Palomo G.; Gómez-Pozos H.; Bernal J. L. Fabrication and Characterization of CdS Thin Film Synthesized by CBD Deposited from pH-Controlled Growth Solutions for Solar Cells Applications. Metallogr. Microstruct. Anal. 2016, 5, 62–68. 10.1007/s13632-015-0253-x.

[ref73] Bube R. H. Photovoltaic Cells and Phenomena. Photovoltaic Materials, 1st ed.; Newman R. C., Ed.; Imperial College Press: London, UK, 1998; Vol 1, ( (6), ).

[ref74] Khimani A. J.; Chaki S. H.; Malek T. J.; Tailor J. P.; Chauhan S. M.; Deshpande M. P.; Tailor; Jiten P.; Chauhan; Sanjaysinh M.; Deshpande M. P. Cadmium sulphide (CdS) thin films deposited by chemical bath deposition (CBD) and dip coating techniques—a comparative study. Mater. Res. Express 2018, 5 (3), 036406 10.1088/2053-1591/aab28d.

[ref75] Bhandari K. P.; Roland P. J.; Mahabaduge H.; Haugen N. O.; Grice C. R.; Jeong S.; Dykstra T.; Gao J.; Ellingson R. J. Thin film solar cells based on the heterojunction of colloidal PbS quantum dots with CdS. Energy Mater. Sol. Cells 2013, 117, 476–482. 10.1016/j.solmat.2013.07.018.

[ref76] Hernández-Borja J.; Vorobiev Y. V.; Ramíez-Bon R. Thin film solar cells of CdS/PbS chemically deposited by an ammonia-free process Solar. Energy Mater. Sol. Cells 2011, 95, 1882–1888. 10.1016/j.solmat.2011.02.012.

[ref77] Mohamed H. A. Theoretical study of the efficiency of CdS/PbS thin film solar cells. Sol. Energy 2014, 108, 360–369. 10.1016/j.solener.2014.07.017.

[ref78] Bhandari K. P.; Roland P. J.; Mahabaduge H.; Haugen N. O.; Grice C. R.; Jeong S.; Dykstra T.; Gao J.; Ellingson R. J. Thin film solar cells based on the heterojunction of colloidal PbS quantum dots with CdS. Sol. Energy Mater. Sol. Cells 2013, 117, 476–482. 10.1016/j.solmat.2013.07.018.

[ref79] Pérez-García C. E.; Meraz-Dávila S.; Chávez-Urbiola E. A.; Chávez-Urbiola I. R.; Willars-Rodríguez F.; Ramírez-Bon R.; Vorobiev Y. Short Communication Chemical Deposition of ITO/CdS/PbS/C for Low Voltage Photosensor Applications. Int. J. Electrochem. Sci. 2018, 13, 3452–3459. 10.20964/2018.04.22.

[ref80] Popescu V.; Nascu H. I.; Darvasi E. Optical properties of PbS-CdS multilayers and mixed (CdS+PbS) thin films deposited on glass substrate by spray pyrolysis. J. Optoelectron. Adv. Mater. 2006, 8 (3), 1187–1193.

[ref81] Zhao Z.; Wang P.; Fan L.; Chen Z.; Shen Z. Design and characterization of PbS/CdS based photovoltaic cell. Adv. Mater. Res. 2013, 820, 7–10. 10.4028/www.scientific.net/AMR.820.7.

[ref82] Onyia A. I.; Mabuchi M. N. Study of optical properties of CdS/PbS and PbS/CdS heterojunction thin films deposited using solution growth technique. Chalcogenide Lett. 2014, 11 (9), 443–452.

[ref83] Kavitha N.; Chandramohan R.; Valanarasu S.; Vijayan T. A.; Rosario S. R.; Kathalingam A. Effect of film thickness on the solar cell performance of CBD grown CdS/PbS heterostructure. J. Mater. Sci.: Mater. Electron. 2015, 27, 2574–2580. 10.1007/s10854-015-4060-6.

[ref84] Mohammed M. K. A. Studying the Structural, Morphological, Optical, and Electrical Properties of CdS/PbS Thin Films for Photovoltaic Applications. Plasmonics 2020, 15, 1989–1996. 10.1007/s11468-020-01224-5.

[ref85] Onyia A. I. Heat Treatment Effects on Properties of CdS/PbS Heterojunction Thin Nanofilms. Int. J. Eng. Invest. 2020, 9 (103), 65–70.

[ref86] Rosario S. R.; Kulandaisamy I.; Arulanantham A. M. S.; Deva Arun Kumar K.; Valanarasu S.; Shkir M.; Kathalingam A.; AlFaify S. Fabrication and characterization of lead sulfide (PbS) thin film based heterostructure (FTO/CdS/PbS/Ag) solar cell by nebulizer spray method. Mater. Res. Express 2019, 6 (5), 056416 10.1088/2053-1591/ab0593.

[ref87] Pérez-García C. E.; Meraz-Dávila S.; Arreola-Jardón G.; de Moure-Flores F.; Ramírez-Bon R.; Vorobiev Y. V. Characterization of PbS films deposited by successive ionic layer adsorption and reaction (SILAR) for CdS/PbS solar cells application. Mater. Res. Express 2020, 7 (1), 015530 10.1088/2053-1591/ab6b5c.

[ref88] Göde F.; Ünlü S. Synthesis and characterization of CdS window layers for PbS thin film solar cells. Mater. Sci. Semicond. Process. 2019, 90, 92–100. 10.1016/j.mssp.2018.10.011.

[ref89] Orozco-Terán R.; Sotelo-Lerma M.; Ramirez-Bon R.; Quevedo-López M.; Mendoza-González O.; Zelava-Angel O. Pbs-Cds Bilayers Prepared by the Chemical Bath Deposition Technique at Different Reaction Temperatures. Thin Solid Films 1999, 343–344, 587–590. 10.1016/S0040-6090(98)01719-2.

[ref90] Saikia D.; Phukan P. Fabrication and evaluation of CdS/PbS thin film solar cell by chemical bath deposition technique. Solid Films 2014, 562, 239–243. 10.1016/j.tsf.2014.04.065.

[ref91] Encinas-Terán A. Obtención de una celda fotovoltaica utilizando CdS y PbS como capas activas, Tesis Ingeniero Químico, 2022.

